# State of Urine in Hepatic Disease

**Published:** 1864-06

**Authors:** 


STATE OF URINE IN HEPATIC DISEASE.—Dr. Eiselt, of Prauge, has called attention to the fact that in cases of melanotic cancer of the liver, the true nature of the affection may be sometimes discovered during life, by the presence of melanine in the patient's urine. Such urine, when left for some hours exposed to the air, becomes of a dark hue, even as dark as porter. Frerichs has shown that the two substances tyrosine and leucine, which were formerly only known to the scientific chemist, are invariably to be found in the urine of patients laboring under acute or yellow atrophy of the liver.—*Braithwaite.*

